# T Cell–Derived IL-10 Impairs Host Resistance to *Mycobacterium tuberculosis* Infection

**DOI:** 10.4049/jimmunol.1601340

**Published:** 2017-06-05

**Authors:** Lúcia Moreira-Teixeira, Paul S. Redford, Evangelos Stavropoulos, Nico Ghilardi, Craig L. Maynard, Casey T. Weaver, Ana Paula Freitas do Rosário, Xuemei Wu, Jean Langhorne, Anne O’Garra

**Affiliations:** * Laboratory of Immunoregulation and Infection, The Francis Crick Institute, London NW1 1AT, United Kingdom;; † Department of Immunology, Genentech Inc., South San Francisco, CA 94080;; ‡ Department of Pathology, University of Alabama at Birmingham, Birmingham, AL 35294;; § Malaria Immunology Laboratory, The Francis Crick Institute, London NW1 1AT, United Kingdom; and; ¶ National Heart and Lung Institute, Faculty of Medicine, Imperial College London, London SW3 6NP, United Kingdom

## Abstract

Tuberculosis (TB), caused by *Mycobacterium tuberculosis* infection, is a leading cause of mortality and morbidity, causing ∼1.5 million deaths annually. CD4^+^ T cells and several cytokines, such as the Th1 cytokine IFN-γ, are critical in the control of this infection. Conversely, the immunosuppressive cytokine IL-10 has been shown to dampen Th1 cell responses to *M. tuberculosis* infection impairing bacterial clearance. However, the critical cellular source of IL-10 during *M. tuberculosis* infection is still unknown. Using IL-10 reporter mice, we show in this article that during the first 14 d of *M. tuberculosis* infection, the predominant cells expressing IL-10 in the lung were Ly6C^+^ monocytes. However, after day 21 postinfection, IL-10–expressing T cells were also highly represented. Notably, mice deficient in T cell–derived IL-10, but not mice deficient in monocyte-derived IL-10, showed a significant reduction in lung bacterial loads during chronic *M. tuberculosis* infection compared with fully IL-10–competent mice, indicating a major role for T cell–derived IL-10 in TB susceptibility. IL-10–expressing cells were detected among both CD4^+^ and CD8^+^ T cells, expressed high levels of CD44 and Tbet, and were able to coproduce IFN-γ and IL-10 upon ex vivo stimulation. Furthermore, during *M. tuberculosis* infection, *Il10* expression in CD4^+^ T cells was partially regulated by both IL-27 and type I IFN signaling. Together, our data reveal that, despite the multiple immune sources of IL-10 during *M. tuberculosis* infection, activated effector T cells are the major source accounting for IL-10–induced TB susceptibility.

## Introduction

Tuberculosis (TB) remains a major threat to global health, with currently one third of the population being infected with *Mycobacterium tuberculosis*, leading to ∼1.5 million deaths annually (1). The control and clearance of this intracellular pathogen rely on the induction of several cytokines during infection, such as IL-12, IFN-γ, or TNF (2–5). Indeed, mutations in the IL-12 and IFN-γ signaling pathways or TNF neutralization, the latter used to treat rheumatoid arthritis or Crohn’s disease, are strongly associated with increased susceptibility to mycobacterial disease (6–10). The critical role of IL-12, IFN-γ, and TNF has also been demonstrated in mouse models of *M. tuberculosis* infection. Mice deficient in IL-12 (11–13), IFN-γ (14, 15), or TNF (16) are not able to build an effective immune response against *M. tuberculosis* and rapidly succumb to infection. IL-12, produced by APC early during infection, stimulates the differentiation and activation of CD4^+^ Th1 cells to release IFN-γ (11–13). In turn, IFN-γ activates macrophages to produce TNF and other proinflammatory cytokines, which in combination with IFN-γ promote *M. tuberculosis* killing through the production of reactive oxygen and nitrogen species (14–17).

Conversely, the immunosuppressive cytokine IL-10 has been reported to limit the protective immune response to *M. tuberculosis* infection, contributing to increased susceptibility to TB (18). In humans, active TB correlates with increased levels of IL-10 (19–23). IL-10 has been shown to be elevated in the pleural fluid (19, 23), bronchoalveolar lavage fluid (BALF) (22), sputum (23), and serum (20, 21) of patients with active pulmonary TB (PTB) compared with healthy controls or patients with other nonmycobacterial diseases. Moreover, T cell proliferation (24) and IFN-γ production (24, 25) from PBMCs obtained from PTB patients have been shown to be impaired in response to *M. tuberculosis* stimulation by endogenous IL-10. Production of IL-10 by human macrophages infected with *M. tuberculosis* has also been shown to inhibit phagosome maturation, resulting in impaired bacterial clearance (26).

Infection of both genetically resistant (C57BL/6 and BALB/c) and susceptible (CBA/J) mice with a common laboratory strain of *M. tuberculosis* (H37Rv or Erdman) induces detectable levels of *Il10* mRNA in the lungs within the first 3–4 wk postinfection (27–29), although higher levels of IL-10 were detected in the lungs of susceptible mice during chronic infection (27). Early studies using IL-10–deficient mice were inconclusive about the functional role of IL-10 during *M. tuberculosis* infection (28, 30, 31), but more recent studies have shown that IL-10 plays a detrimental role during infection by limiting host-protective immune responses (18, 29, 32, 33). Resistant and susceptible mice either deficient in IL-10 (18, 33) or treated with blocking Abs to neutralize IL-10 action (32–34) showed enhanced protection against *M. tuberculosis* infection. Decreased bacterial loads in the absence of IL-10 correlated with early and enhanced production of cytokines associated with protection (e.g., IFN-γ, TNF, and GM-CSF) and increased influx of CD4^+^ Th1 cells into the lungs of *M. tuberculosis*–infected mice (29). Further evidence for a detrimental role of IL-10 during *M. tuberculosis* infection arose from the findings that overexpression of IL-10 increases host susceptibility to TB by limiting Th1 cell responses and macrophage bactericidal functions (27, 35).

IL-10 can be produced by almost all cell types of both the innate (e.g., macrophages, monocytes, neutrophils, dendritic cells [DCs], NK cells) and adaptive (e.g., T and B cells) immune response (36). To date, there is limited information on the specific cellular sources of IL-10 during the course of *M. tuberculosis* infection and their relative contribution to host susceptibility to TB (reviewed in Refs. 5, 18). In humans, monocytes isolated from PTB patients have been shown to produce higher levels of IL-10 than monocytes from healthy controls (37). In mice, overexpression of IL-10 by macrophages and monocytes (under control of the CD68 promoter) has been shown to impair macrophage function during *M. tuberculosis* infection, increasing host susceptibility to TB (35). However, IL-10 production during *M. tuberculosis* infection does not seem to be restricted to myeloid cells. Human CD4^+^ T cells isolated from the BALF of active PTB patients have been reported to produce both IFN-γ and IL-10 in response to mycobacterial Ags (38). Furthermore, overexpression of IL-10 by activated T cells (under control of the IL-2 promotor) during *M. tuberculosis* infection has been shown to enhance mice susceptibility to TB by limiting Th1 cell responses (27). However, systematic studies detailing the specific cellular sources of IL-10 during *M. tuberculosis* infection that are not reliant on overexpression systems have not been forthcoming. This may be in part because of the low expression and inherent instability of IL-10 (39), which makes its detection by conventional assays challenging.

Using IL-10 reporter mice, we show in this article that IL-10 expression is detected early predominantly in Ly6C^+^ monocytes and after day 21 postinfection in T cells. Increased control of *M. tuberculosis* infection was observed in T cell–specific *Il10*-deficient mice, closely resembling the phenotype observed in complete *Il10*-deficient mice, indicating that T cells are the critical source of IL-10–induced TB susceptibility. Although many different immune cells produced IL-10 during *M. tuberculosis* infection, we demonstrated that disease susceptibility was mainly driven by IL-10 derived from activated effector T cells, and its expression was enhanced by IL-27 and type I IFN signaling.

## Materials and Methods

### Mice

C57BL/6 wild-type (WT), IL-10–deficient (*Il10^−/−^*) (40), IL-27Rα–deficient (*Tccr^−/−^*, referred to as *Il27ra^−/−^* in this article; from Genentech, South San Francisco, CA) (41), type I IFN receptor–deficient (*Ifnar1^−/−^*) (42) mice, and “*Il10* BAC-in transgene” (10BiT) IL-10 reporter mice (43) were bred and housed in specific pathogen-free facilities at The Francis Crick Institute, Mill Hill Laboratory (London, U.K.). *Il10^flox/flox^* (*Il10^fl/fl^*) mice, which have loxP sites flanking exon 1 of *Il10* (44), crossed with CD4-Cre (44, 45), LysM-Cre (46, 47), CD11c-Cre (48), and CD19-Cre (49) mice and backcrossed for 10 generations onto the C57BL/6 background (50), were also bred and housed in specific pathogen-free facilities at The Francis Crick Institute, Mill Hill Laboratory. Littermate control (*Il10^fl/fl^* Cre^−^) mice were used in all experiments. Female mice were used between 8 and 16 wk of age. All protocols for breeding and experiments were performed in accordance with Home Office (U.K.) requirements and the Animal Scientific Procedures Act, 1986.

### Experimental infection

*M. tuberculosis* experiments were performed under BSL-3 conditions. *M. tuberculosis* HN878 bacilli were grown to midlog phase in Middlebrook 7H9 broth supplemented with 10% oleic acid albumin dextrose complex (Difco), 0.05% Tween 80, and 0.5% glycerol before being quantified on 7H11 agar plates and stored in aliquots at −80°C. Mice were infected via the aerosol route using a three-jet Collison nebulizer unit (BGI), calibrated to deliver ∼100–200 CFUs to the lung. The infection dose was confirmed by determining the number of viable bacteria in the lungs of five mice just after the aerosol infection. For bacterial load determination, mice were euthanized by CO_2_ inhalation and the lungs were aseptically excised, individually homogenized, as described previously (29), followed by plating serial dilution of the organ homogenate on Middlebrook 7H11 agar supplemented with 10% oleic acid albumin dextrose complex. CFUs were counted after 3 wk of incubation at 37°C, and the bacterial load per organ was calculated.

### Flow cytometry

To track *Il10* (Thy1.1) expression in the lungs of 10BiT IL-10 reporter mice during *M. tuberculosis* infection, we prepared single-cell homogenates, as described previously (29), washed in PBS (Life Technologies) and stained according to manufacturer’s instructions to exclude dead cells using a Live/Dead fixable red dead cell stain kit (Invitrogen). Cells were pretreated for 10 min with anti-FcgRI/FcgRII (anti-CD16/CD32) Ab. Cells were then stained with anti-Thy1.1 (HIS51; eBioscience) and other Abs against the following extracellular markers to identify myeloid cells and lymphocytes, as described previously (50, 51). Myeloid cell markers included Ly6G (1A8; BD), Ly6C (HK1.4; eBioscience), Thy1.2 (53-2.1; eBioscience), CD11c (HL3; BD), CD11b (M1/70; BD), F4/80 (BM8; eBioscience), and MHC class II (M5/114.15.2; eBioscience). Lymphoid cell markers included Thy1.2 (53-2.1; eBioscience), CD3 (145-2C11; eBioscience), CD4 (RM4-5; eBioscience/BD), CD8 (53-6.7; eBioscience), γδ TCR (GL3; eBioscience), and CD19 (eBio1D3 [eBioscience]; 6D5 [BioLegend]). In some experiments, anti-CD44 (1M7; eBioscience) Ab was also used. For intranuclear transcription factor expression, cells were stained with anti-Foxp3 (FJK-16s; eBioscience) and anti-Tbet (4B10; BioLegend) Abs using the Foxp3/Transcription Factor Staining Buffer Set (eBioscience) according to the manufacturer’s instructions. Isotype control eBR2a (eBioscience) and MOPC-21 (BioLegend) were used as negative control. For cytokine analysis, cells were restimulated ex vivo with *M. tuberculosis* tuberculin purified protein derivative (PPD; 20 μg/ml; Statens Serum Institute) and anti-CD28 (2 μg/ml, clone 37.51; Harlan) for 20 h. Brefeldin A (10 μg/ml; Sigma-Aldrich) was added during the last 4 h. After extracellular staining, cells were fixed and treated with permeabilization buffer (BD) according to manufacturer’s instructions and stained with anti–IFN-γ (XMG1.2; eBioscience) or isotype control (eBRG1; eBioscience) Abs. All stained samples were fixed with stabilizing fixative (BD) and refrigerated in the dark overnight before being acquired on a CyAN ADP analyzer (Dako, Ely, U.K.) using Summit software (Cytomation). Data were analyzed using FlowJo software (Tree Star).

### Quantitative real time-PCR

CD4^+^ cells from infected WT, *Il27ra^−/−^*, and *Ifnar1^−/−^* mice were enriched from lung homogenates using anti-CD4 microbeads (L3T4; Miltenyi Biotec) according to manufacturer’s instructions. Purified cells were >95% CD4^+^ T cells as assessed by flow cytometry (data not shown). Cells were kept in 350 μl of RLT buffer (Qiagen) at −80°C before processing. RNA was extracted using RNeasy Mini Kits (Qiagen) and reverse transcribed to cDNA with a high-capacity reverse transcription kit (Applied Biosystems). The expression of indicated genes was quantified by real-time PCR (ABI Prism 7900; Applied Biosystems) and normalized against *Hprt1* mRNA levels. TaqMan primer probes (Applied Biosystems) for *Il10* (Mm00439616_m1), *Ifng* (Mm01168134_m1), *Csf2* (Mm01290062_m1), and *Hprt1* (Mm00446968_m1) were used.

### Statistics

Data are shown as the mean ± SEM. Statistical tests, as described in the figure legends, were used to compare experimental groups, with *p* < 0.05 considered significant. GraphPad Prism 6 (GraphPad Software) was used for data analysis and preparation of all graphs.

## Results

### Immune sources of IL-10 during *M. tuberculosis* infection

The specific cellular sources of IL-10 during *M. tuberculosis* infection that can contribute to TB susceptibility are still undetermined. To address this issue and track cell-specific expression of IL-10, we used a reporter mouse that, through the expression of the surface marker Thy1.1, stably identifies all cells in which *Il10* alleles have been activated [10BiT reporter mice, described previously (43); hereafter simply IL-10 reporter mice]. Multiparameter flow cytometry analyses were performed at different times postinfection with the hypervirulent W-Beijing *M. tuberculosis* strain HN878, which was used for this study because it has been reported to induce high levels of IL-10 (52). IL-10 expression in 10BiT reporter mice was determined by comparing its staining profile with that seen in WT C57BL/6 control mice, which lack the Thy1.1 expression cassette (Supplemental Fig. 1A). Postinfection with *M. tuberculosis* HN878, there was an early increase in the percentage and number of IL-10^+^ cells detectable in the lungs, which peaked around days 21–28 postinfection (Supplemental Fig. 1B).

**FIGURE 1.**
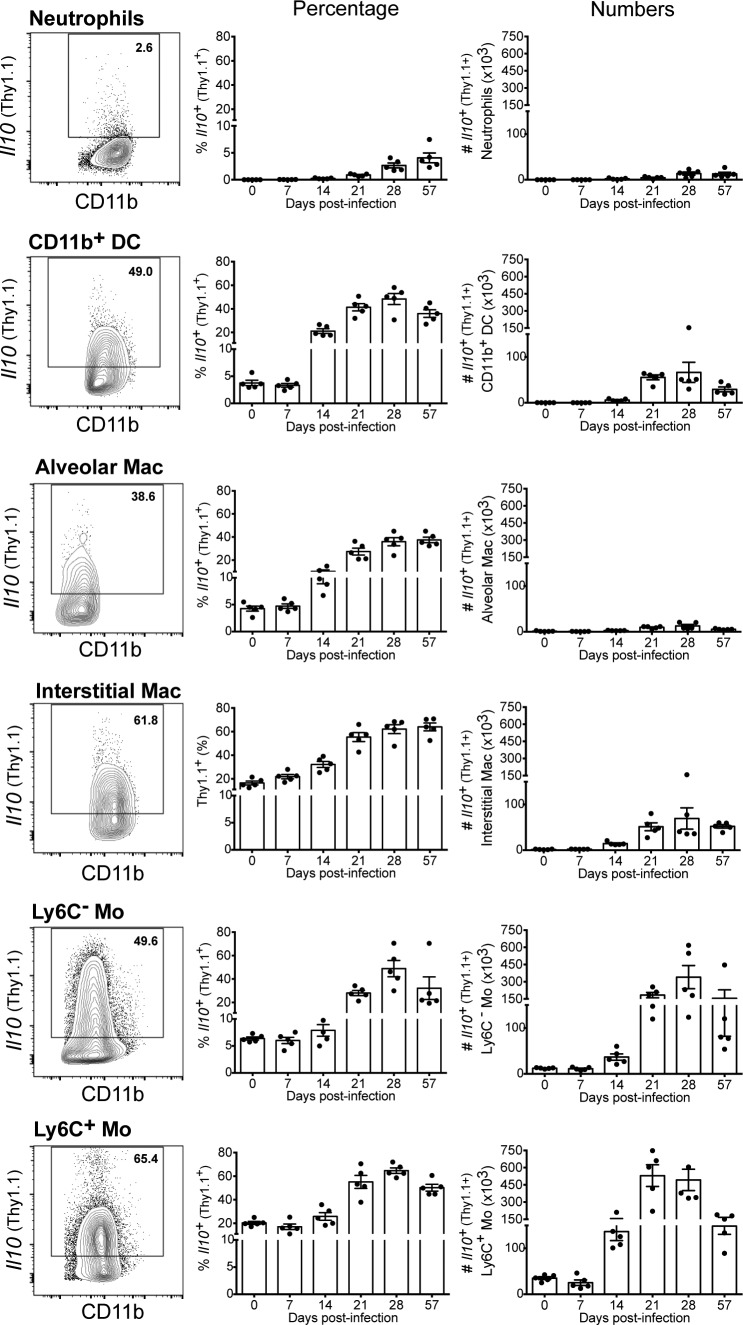
Lung monocytes, macrophages, and DCs express IL-10 during *M. tuberculosis* infection. 10BiT IL-10 reporter mice were infected with *M. tuberculosis* HN878. At indicated days postinfection, lung cell suspensions were prepared and stained as described in the *Materials and Methods*. Myeloid cells, gated as shown in Supplemental Fig. 1C, were analyzed for the expression of *Il10* (Thy1.1^+^). Flow cytometry plots show concatenated data of five lungs at day 28 postinfection from one representative experiment (left panel). The percentage (middle panel) and the total number (right panel) of *Il10* (Thy1.1)-expressing cells in the lungs for each indicated population are shown as mean ± SEM. Results are representative of three or more independent experiments with individual data points depicting individual mice (three to five mice per time point per experiment). Mac, macrophages; Mo, monocytes.

To elucidate which cells are the key sources of IL-10 during *M. tuberculosis* HN878 infection, we assessed the expression of IL-10 among different lung myeloid cell populations (Fig. 1), identified as described in Supplemental Fig. 1C. Neutrophils expressed very low levels of IL-10 at the steady-state (day 0) and throughout the course of infection, whereas a small proportion (5–20%) of CD11b^+^ DCs, alveolar and interstitial macrophages, and Ly6C^−^ and Ly6C^+^ monocytes expressed detectable levels of IL-10 already at the steady-state, which greatly increased with infection (Fig. 1). Although the frequency of IL-10^+^ cells among these myeloid cells did not increase over the first few days postinfection, between days 7 and 28 postinfection, the frequency of IL-10–expressing cells increased 2- to 3-fold among Ly6C^+^ monocytes and interstitial macrophages, and 8- to 10-fold among Ly6C^−^ monocytes, alveolar macrophages, and CD11b^+^ DCs (Fig. 1). Despite this increased frequency in IL-10^+^ cells, relatively low numbers of alveolar and interstitial macrophages and CD11b^+^ DCs expressing IL-10 were detected in the lungs throughout infection (<1 ×10^5^ cells per lung) (Fig. 1). In contrast, IL-10–producing Ly6C^−^ and Ly6C^+^ monocytes were detected in large numbers in the lungs after 21 d postinfection (up to 3.40 ± 1.01 × 10^5^ and 5.29 ± 0.95 × 10^5^ cells per lung, respectively) (Fig. 1).

**FIGURE 2.**
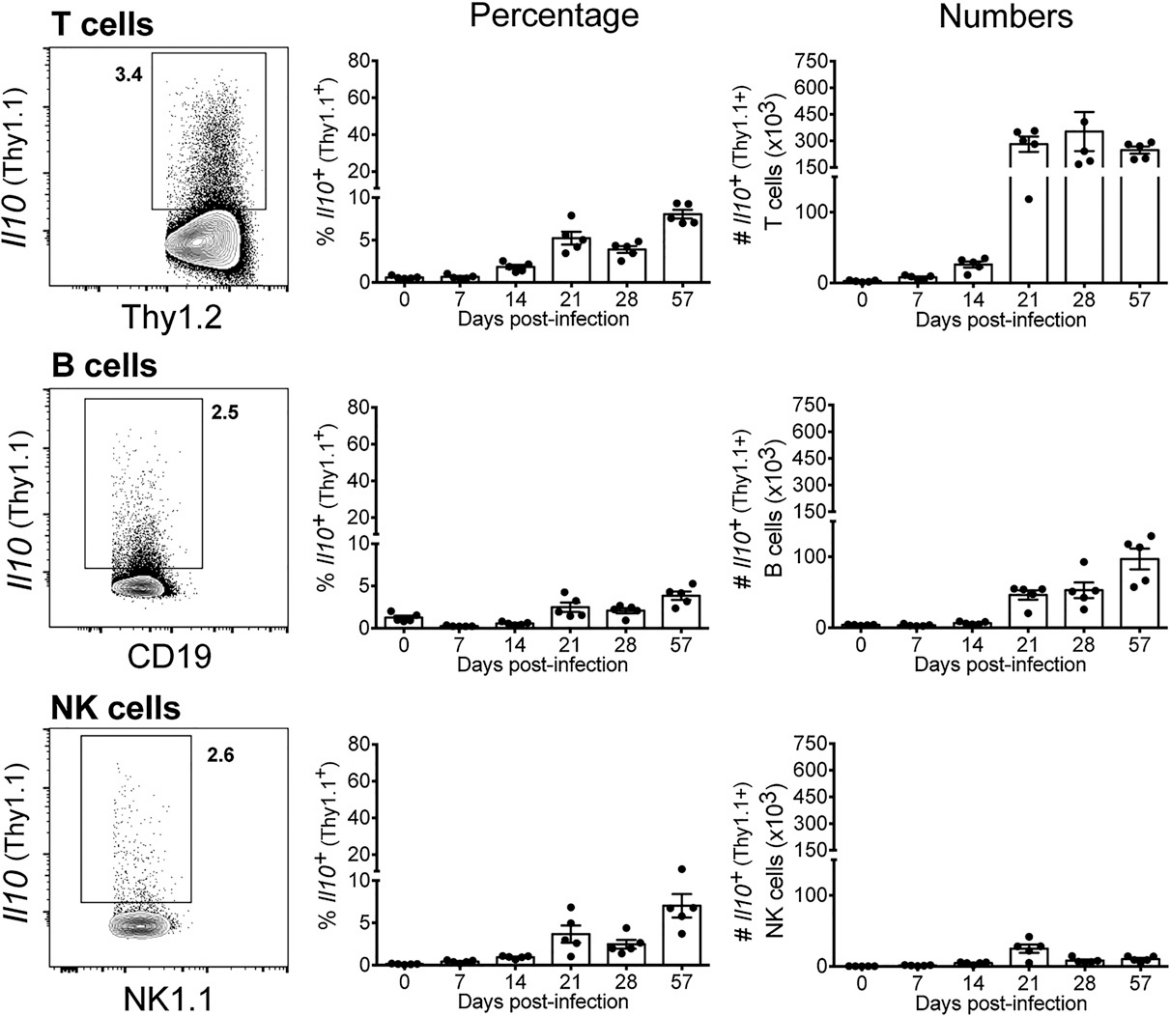
IL-10–expressing T cells are detected in high numbers in the lung during the adaptive immune response to *M. tuberculosis* infection. 10BiT IL-10 reporter mice were infected with *M. tuberculosis* HN878. At indicated days postinfection, lung cell suspensions were prepared and stained as described in the *Materials and Methods*. Lymphoid cells, gated as shown in Supplemental Fig. 1D, were analyzed for the expression of *Il10* (Thy1.1^+^). Flow cytometry plots show concatenated data of five lungs at day 28 postinfection from one representative experiment (left panels). The percentage (middle panels) and the total number (right panels) of *Il10* (Thy1.1)-expressing cells in the lungs for each indicated population are shown as mean ± SEM. Results are representative of three or more independent experiments with individual data points depicting individual mice (three to five mice per time point per experiment).

We then sought to determine the expression of IL-10 among different lung lymphoid cells (Fig. 2), identified as described in Supplemental Fig. 1D. T, B, and NK cells expressing IL-10 were barely detected at the steady-state and during the first 2 wk of infection, but were consistently detected after 21 d postinfection (Fig. 2), although at lower frequencies compared with those among myeloid cells (Fig. 1). This increase in frequency was accompanied by a great increase in the numbers of IL-10^+^ T and B cells detected in infected lungs between days 14 and 21 postinfection. After 21 d postinfection, a large number of IL-10–expressing T cells was detected in infected lungs (up to 3.53 ± 1.11 × 10^5^ per lung), whereas IL-10–expressing B cells were detected at lower numbers at all time points analyzed (from 0.46 ± 0.07 × 10^5^ to 0.97 ± 0.15 × 10^5^ cells per lung) (Fig. 2). In contrast, IL-10^+^ NK cell numbers remained very low throughout infection (<0.4 × 10^5^ cells per lung) (Fig. 2). Together, these results suggest that both monocytes and T cells might be the dominant sources of IL-10 during *M. tuberculosis* infection.

### T cell–specific IL-10 deficiency increases host protection against *M. tuberculosis*, reproducing the phenotype observed in *Il10^−/−^* mice

**FIGURE 3.**
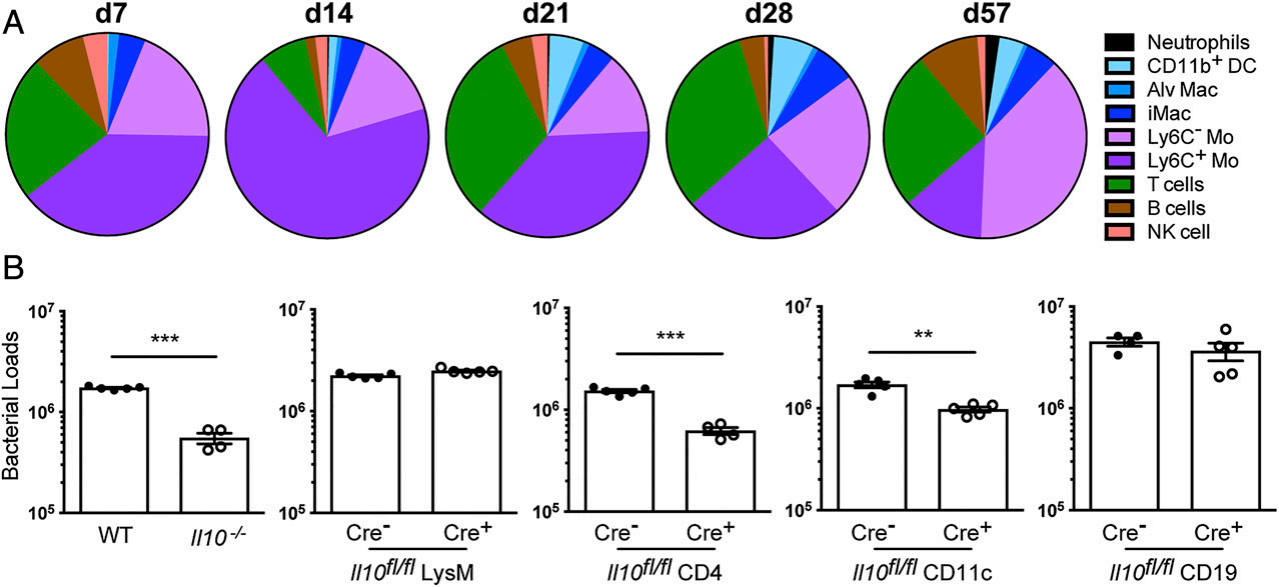
Target deletion of *Il10* in T cells increases bacterial clearance during *M. tuberculosis* infection. (**A**) 10BiT IL-10 reporter mice were infected with *M. tuberculosis* HN878. At indicated days postinfection, lung cells were analyzed for the expression of *Il10* (Thy1.1^+^) as shown in Figs. 1 and 2. Pie charts represent the distribution of indicated cell subsets among total *Il10^+^* (Thy1.1^+^) cells at indicated days postinfection. (**B**) WT, *Il10*^−/−^, *Il10^fl/fl^* LysM-Cre^+^, *Il10^fl/fl^* CD4-Cre^+^, *Il10^fl/fl^* CD11c-Cre^+^, and *Il10^fl/fl^* CD19-Cre^+^ mice and respective Cre^−^ littermate controls were infected with *M. tuberculosis* HN878. Bacterial loads in the lungs were determined after 60 d of infection. Data show the mean ± SEM of one representative out of five for the *Il10*^−/−^, three for the *Il10^fl/fl^* LysM-Cre^+^, three for the *Il10^fl/fl^* CD4-Cre^+^, three for the *Il10^fl/fl^* CD11c-Cre^+^, and two for the *Il10^fl/fl^* CD19-Cre^+^ mice; independent experiments with four to five mice per group per experiment. Differences were tested for significance by an unpaired Student *t* test. **p < 0.01, ***p < 0.001. Alv Mac, alveolar macrophages; iMac, interstitial macrophages; Mo, monocytes.

We showed that during *M. tuberculosis* infection, IL-10 can be produced by cells of both the innate and the adaptive immune systems (Figs. 1, 2). During the innate phase of the immune response to *M. tuberculosis* infection, monocytes (mainly Ly6C^+^ monocytes) were clearly the biggest IL-10^+^ population, accounting for up to 70% of total IL-10–expressing cells in the lungs by day 14 postinfection (Fig. 3A). From day 21 postinfection onward, monocytes accounted for nearly 50% of total IL-10^+^ cells in the lungs followed by T cells that account for nearly 25%, as shown in Fig. 3A. Other cell types represented only minor sources of IL-10 throughout infection (Fig. 3A).

To determine the impact of cell-specific IL-10 on disease outcome, we evaluated whether selective deletion of *Il10* in LysM^+^ cells (monocytes, macrophages, and neutrophils), CD11c^+^ cells (mostly DCs and macrophages), T cells, or B cells could reproduce the phenotype observed in fully deficient *Il10^−/−^* mice. In Supplemental Fig. 2 we show that *Il10* mRNA levels are significantly diminished/almost abrogated in all of the respective specific cell types from *Il10^fl/fl^* LysM-Cre^+^, *Il10^fl/fl^* CD11c-Cre^+^, *Il10^fl/fl^* CD4-Cre^+^, and *Il10^fl/fl^* CD19-Cre^+^ mice, but not the Cre^−^ control mice, whereas *Tnf* or *Ifng* mRNA expression was unaffected. To determine the effect of cell-specific deletion on the outcome of *M. tuberculosis* infection, we infected WT, *Il10^−/−^*, *Il10^fl/fl^* LysM-Cre^+^, *Il10^fl/fl^* CD11c-Cre^+^, *Il10^fl/fl^* CD4-Cre^+^, and *Il10^fl/fl^* CD19-Cre^+^ mice and respective Cre^−^ littermate controls with *M. tuberculosis* HN878 (Fig. 3B). At day 60 postinfection, we observed up to 70% inhibition of bacterial growth in the lungs of *Il10^−/−^* mice compared with WT control mice (Fig. 3B), reproducing our own earlier findings with H37Rv (29), determining the time point for further comparative experiments. Although monocytes were the largest IL-10^+^ population detected in infected lungs throughout infection, IL-10 from these cells did not appear to impair host resistance to *M. tuberculosis* infection because similar bacterial loads were detected in the lungs of *Il10^fl/fl^* LysM-Cre^+^ and their Cre^−^ littermate controls (Fig. 3B). In contrast, mice deficient in T cell–derived IL-10 (*Il10^fl/fl^* CD4-Cre^+^) exhibited a significant decrease in lung bacterial loads after 60 d of *M. tuberculosis* infection, with nearly 60% inhibition of bacterial growth compared with Cre^−^ control mice (Fig. 3B). *Il10^fl/fl^* CD11c-Cre^+^ mice presented partial signs of protection, with nearly 35% inhibition of bacterial growth compared with their Cre^−^ controls (Fig. 3B), suggesting that IL-10–producing CD11c^+^ cells, such as DCs and/or alveolar or interstitial macrophages, may also partially limit the control of *M. tuberculosis* infection. B cell–specific derived IL-10 did not affect bacterial clearance because similar bacterial loads were detected in the lungs of *Il10^fl/fl^* CD19-Cre^+^ mice and their Cre^−^ littermate controls (Fig. 3B). Taken together, these results demonstrate a major role for IL-10 produced by the T cell compartment compared with other cell types in IL-10–induced TB susceptibility, although IL-10 derived from CD11c^+^ cells could also contribute, albeit to a lesser extent. No effect was observed on lung pathology in *Il10^−/−^* mouse lungs as compared with WT control mice (data not shown).

### Effector CD4^+^ and CD8^+^ cells are the main source of T cell–derived IL-10 during *M. tuberculosis* infection

**FIGURE 4.**
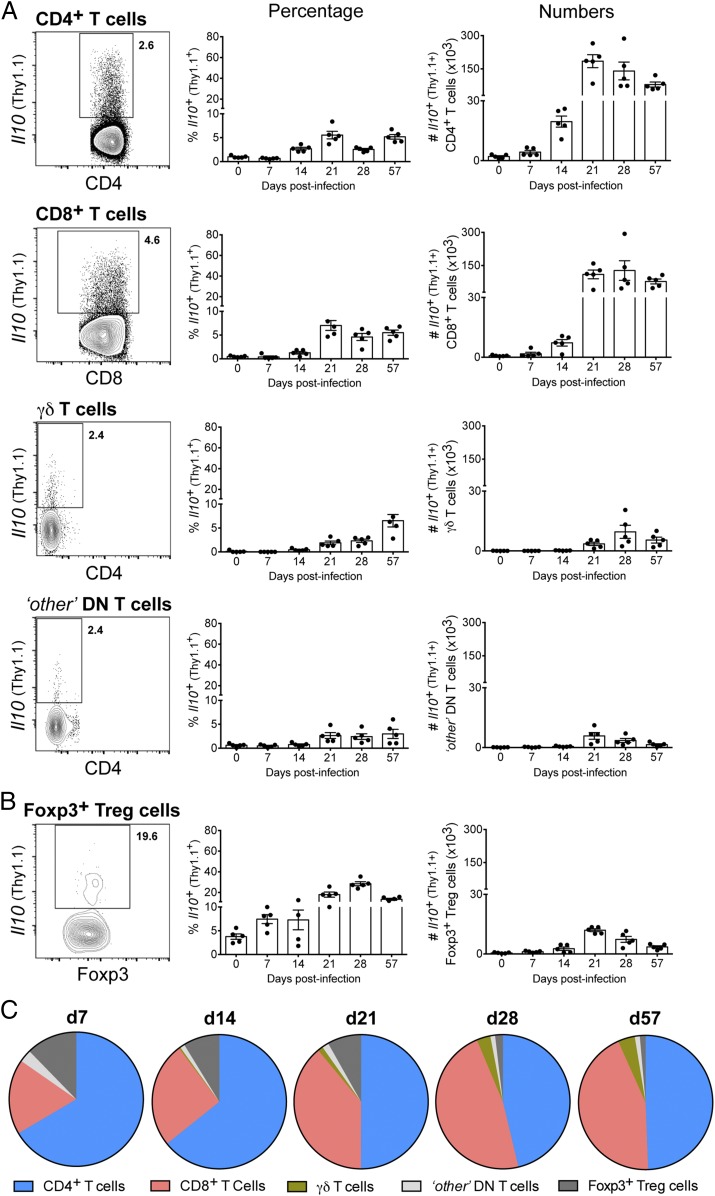
CD4^+^ and CD8^+^ T cells are dominant sources of T cell–derived IL-10 during the adaptive immune response to *M. tuberculosis* infection. (**A** and **B**) 10BiT IL-10 reporter mice were infected with *M. tuberculosis* HN878. At indicated days postinfection, lung T cell subsets (gated as shown in Supplemental Fig. 1D) were analyzed for the expression of *Il10* (Thy1.1^+^). Flow cytometry plots show concatenated data of five lungs at day 28 postinfection from one representative experiment (left panels). The percentage (middle panels) and the total number (right panels) of *Il10* (Thy1.1)-expressing cells in the lungs for each indicated population are shown as mean ± SEM. Results are representative of three (A) or two (B) independent experiments for each time point with individual data points depicting individual mice (three to five mice per time point per experiment). (**C**) Pie charts represent the distribution of indicated T cell subsets among total *Il10^+^* (Thy1.1^+^) T cells at indicated days postinfection.

Because T cells were the major source of IL-10 accounting for impaired host resistance against *M. tuberculosis* infection, we then performed further phenotypic analysis to identify the nature of these cells. We tracked the expression of IL-10 among CD4^+^, CD8^+^ and double-negative T cells in the lungs of 10BiT reporter mice at different times postinfection with *M. tuberculosis* HN878 (Fig. 4A). The different T cell subsets were identified as described in Supplemental Fig. 1D. IL-10 expression was barely detected under steady-state conditions (day 0), but it was consistently detected among all T cell subsets analyzed after days 14 and 21 postinfection (Fig. 4A). Despite the low frequency of IL-10^+^ cells among these T cell subsets, IL-10–producing CD4^+^ and CD8^+^ T cells were detected in large numbers in infected lungs after day 21 postinfection (up to 1.84 ± 0.30 × 10^5^ and 1.28 ± 0.44 × 10^5^ cells per lung, respectively), whereas IL-10–producing γδ T cells and “other” double-negative T cells were detected at lower numbers at all time points analyzed (<0.15 × 10^5^ cells per lung) (Fig. 4A). To determine the contribution of Foxp3^+^ regulatory T (Treg) cells to this high number of IL-10^+^ CD4^+^ T cells detected in infected lungs (Fig. 4A), we performed intracellular staining to detect Foxp3 expression (Supplemental Fig. 1D). Low numbers of IL-10–expressing Foxp3^+^ Treg cells were detected in the lungs throughout infection (<0.15 × 10^5^ cells per lung) (Fig. 4B). Indeed, as shown in Fig. 4C, lung Foxp3^+^ Treg cells represented a minor subset of IL-10^+^ T cells in *M. tuberculosis*–infected lungs, accounting for <5% of IL-10^+^ T cells at the peak of IL-10 expression (day 28 postinfection; Supplemental Fig. 1B). CD4^+^ T cells were clearly the biggest IL-10^+^ population, accounting for 65 to 50% of IL-10^+^ T cells, followed by CD8^+^ T cells, which accounted for up to 40% by day 28 postinfection (Fig. 4C).

**FIGURE 5.**
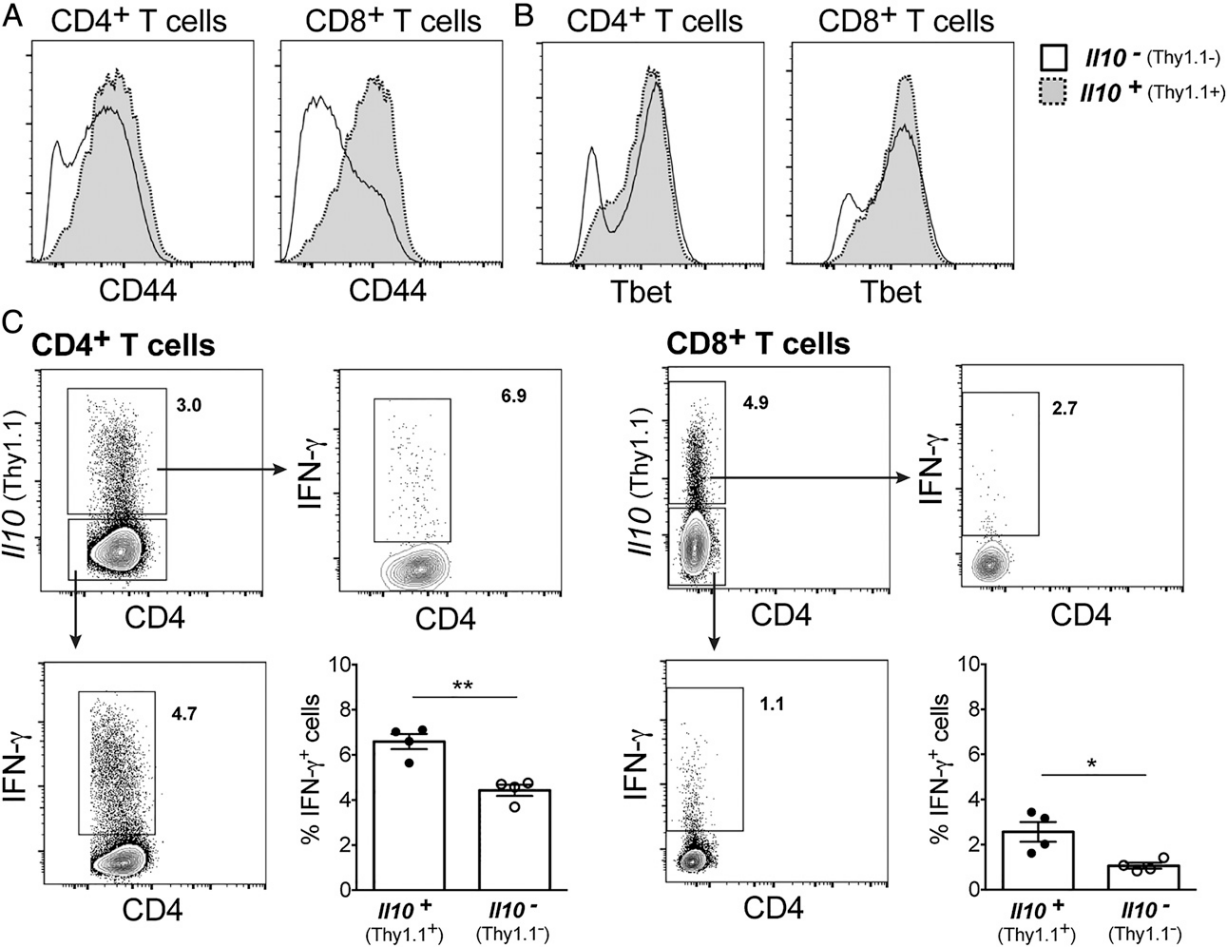
IL-10–expressing T cells from *M. tuberculosis*–infected lungs express Tbet and coproduce IFN-γ. 10BiT IL-10 reporter mice were infected with *M. tuberculosis* HN878. At day 28 postinfection, lung cell suspensions were prepared and stained as described in the *Materials and Methods*. (**A** and **B**) Representative histograms show CD44 surface staining (A) and Tbet intracellular staining (B) for *Il10^+^* (Thy1.1^+^; dashed line, gray) or *Il10^−^* (Thy1.1^−^; solid line, white) CD4^+^ and CD8^+^ T cells. Histograms show concatenated data of five lungs from one representative experiment out of two (for CD8^+^ T cells) or six (for CD4^+^ T cells) independent experiments. (**C**) Production of IFN-γ by *Il10^+^* (Thy1.1^+^) or *Il10^−^* (Thy1.1^−^) cells among CD4^+^ (left panel) or CD8^+^ (right panel) T cells was determined after ex vivo restimulation of whole lung cell homogenates with PPD plus anti-CD28 overnight and brefeldin A for the last 4 h. Plots show concatenated data of four lungs from one representative experiment out of four (for CD8^+^ T cells) or six (for CD4^+^ T cells) independent experiments (four to five mice per experiment). The percentage of IFN-γ–expressing cells among *Il10^+^* (Thy1.1^+^) or *Il10*^−^ (Thy1.1^−^) cells is shown as mean ± SEM, with individual data points depicting individual mice. Differences were tested for significance by an unpaired Student *t* test. *p < 0.05, **p < 0.01.

Further analysis of CD4^+^ and CD8^+^ T cell populations at the peak of IL-10 expression (day 28 postinfection) revealed that IL-10–expressing CD4^+^ and CD8^+^ T cell subsets expressed much higher levels of CD44 than IL-10^−^ cells (Fig. 5A), demonstrating an increased activation state of IL-10^+^ T cells. In addition, the majority of IL-10^+^ cells among CD4^+^ and CD8^+^ T cell subsets expressed high levels of the transcription factor Tbet, higher than their IL-10^−^ counterparts (Fig. 5B). Moreover, IL-10–expressing CD4^+^ and CD8^+^ T cell subsets produced IFN-γ upon ex vivo restimulation (Fig. 5C, Supplemental Fig. 3). Interestingly, higher frequencies of IFN-γ^+^ cells were detected among IL-10–expressing CD4^+^ and CD8^+^ T cells compared with their respective IL-10^−^ counterparts upon ex vivo restimulation with *M. tuberculosis* tuberculin PPD (Fig. 5C). The expression of CD44, Tbet, and IFN-γ by IL-10–expressing CD4^+^ and CD8^+^ T cell subsets suggests that highly activated effector T cells are the major source of T cell–derived IL-10 during *M. tuberculosis* infection.

### IL-27 and type I IFN enhance *Il10* expression in CD4^+^ T cells during *M. tuberculosis* infection

**FIGURE 6.**
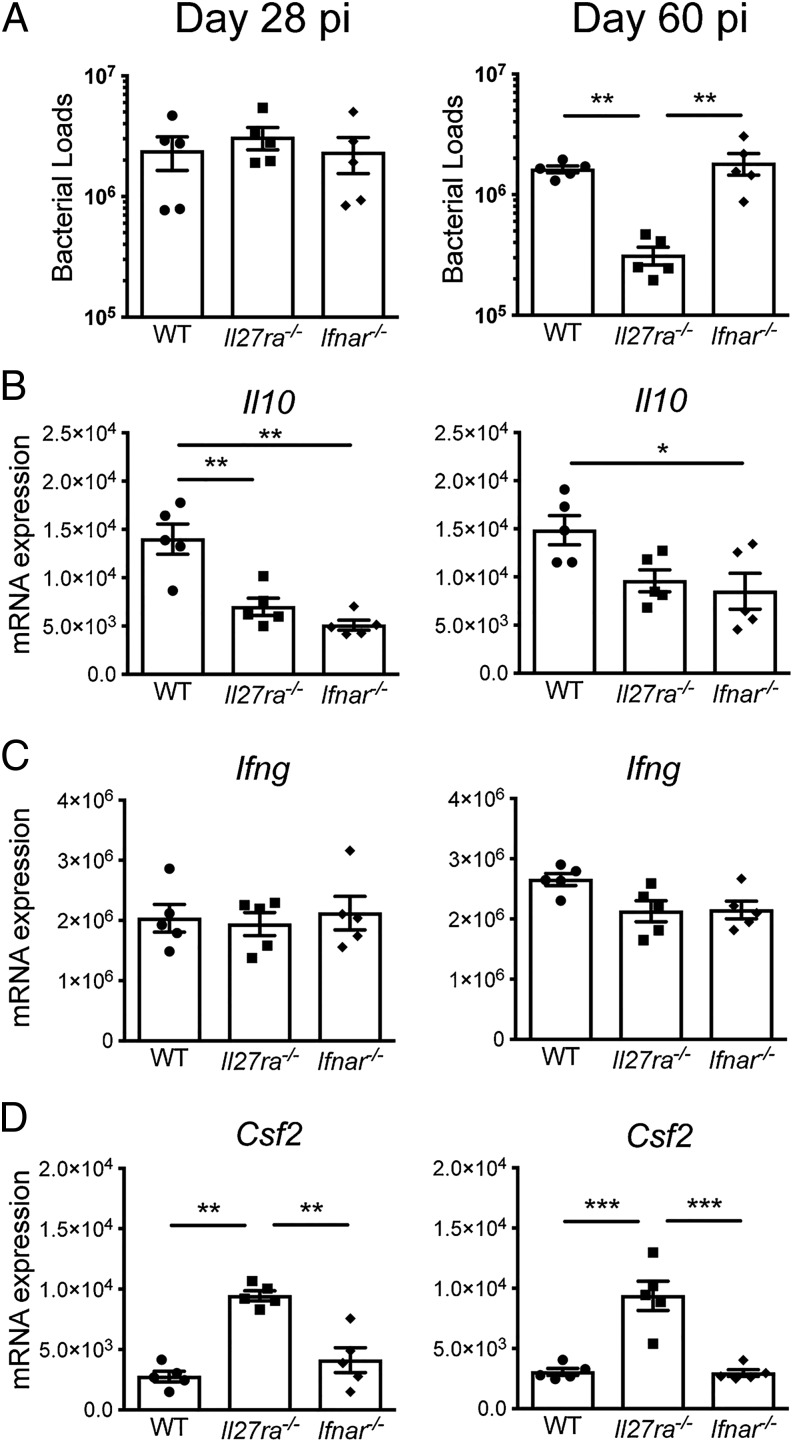
IL-27 and type I IFN signaling regulate *Il10* transcription in lung CD4^+^ T cells during *M. tuberculosis* infection. WT, *Il27ra^−/−^*, and *Ifnar1^−/−^* mice were infected with *M. tuberculosis* HN878. (**A**) Bacterial loads in the lungs were determined after 28 and 60 d of infection. (**B**–**D**) Lung CD4^+^ cells were isolated from infected mice at days 28 (left panels) and 60 (right panels) postinfection. *Il10* (B), *Ifng* (C), and *Cfs2* (D) mRNA expression were analyzed by quantitative real-time PCR and normalized against *Hprt1* mRNA levels. Data show the mean ± SEM of one representative out of two or more independent experiments with three to six mice per group per experiment. Differences were tested for significance by one-way ANOVA. *p < 0.05, **p < 0.01, ***p < 0.001.

IL-27 has been implicated in the regulation of IL-10 production by T cells during infection, such as in leishmaniasis (53) and malaria (50). Although it is still unclear whether IL-27 also regulates IL-10 expression by T cells during *M. tuberculosis* infection, IL-27Rα signaling in CD4^+^ T cells has been recently shown to confer susceptibility to this infection (54). Type I IFN has also been implicated in host susceptibility to TB, and it has been suggested that its pathogenic role during *M. tuberculosis* and other bacterial infections may be linked to the induction of IL-10 (55–58). We therefore evaluated the role of these cytokines in the induction of IL-10 expression in CD4^+^ T cells during *M. tuberculosis* infection. WT, *Il27ra^−/−^*, and *Ifnar1^−/−^* mice were infected with *M. tuberculosis* HN878, and *Il10* mRNA expression in CD4^+^ T cells purified from infected lungs was determined. *Il27ra^−/−^* mice exhibited a significant decrease in lung bacterial loads after 60 d of *M. tuberculosis* infection, in agreement with previous reports (59, 60), as compared with controls. In contrast, similar bacterial loads were detected in the lungs of *Ifnar1^−/−^* mice compared with WT control mice on a C57BL/6 background at days 28 and 60 postinfection (Fig. 6A). *Il10* mRNA expression in purified CD4^+^ T cells was significantly reduced in both *Il27ra^−/−^* and *Ifnar1^−/−^* mice as compared with WT control mice at day 28 postinfection (Fig. 6B, left panel). The expression of *Il10* mRNA remained slightly reduced in CD4^+^ T cells from both *Il27ra^−/−^* and *Ifnar1^−/−^* mice as compared with WT control mice until day 60 postinfection (Fig. 6B, right panel). Despite the lower levels of *Il10* mRNA in the absence of IL-27 and type I IFN signaling, similar levels of *Ifng* mRNA expression were detected in CD4^+^ T cells from WT, *Il27ra^−/−^*, and *Ifnar1^−/−^* mice at days 28 and 60 postinfection (Fig. 6C). CD4^+^ T cells from *Il27ra^−/−^*-infected mice showed increased levels of *Csf2* mRNA (encoding GM-CSF) compared with WT and *Ifnar1^−/−^* mice (Fig. 6D), indicating that IL-27Rα signaling inhibits CD4^+^ T cell expression of GM-CSF, another cytokine implicated in the protective immune response to *M. tuberculosis* infection (61). Our results showed that *Il10* expression in CD4^+^ T cells was partially regulated by IL-27 and type I IFN signaling early during *M. tuberculosis* infection.

## Discussion

IL-10 has been shown to impair immune responses to *M. tuberculosis* infection, contributing to host susceptibility to TB (27, 29, 32, 35). However, it is still unclear which cellular sources of IL-10 are critical for suppression of the immune response against *M. tuberculosis* limiting host protection (5, 18). Using IL-10 reporter mice, in this article we show that IL-10 is expressed by cells from both the innate (predominantly by monocytes) and adaptive (predominantly by T cells) immune response during *M. tuberculosis* infection in vivo. Nevertheless, IL-10–induced susceptibility to *M. tuberculosis* infection was found to be largely dependent on IL-10 derived from activated effector T cells, whose expression during *M. tuberculosis* infection was enhanced by both IL-27 and type I IFN.

To gain new insights into the diversity and distribution of immune cells that express IL-10 during the course of *M. tuberculosis* infection in vivo, we infected IL-10 reporter mice (43) with *M. tuberculosis* HN878. The total number of IL-10^+^ cells in the lungs of *M. tuberculosis*–infected mice greatly increased until day 28 postinfection and then slightly declined as the infection progressed into the chronic phase. This kinetic of IL-10 expression is in line with the levels of IL-10 produced upon ex vivo stimulation of cells isolated from the lungs of *M. tuberculosis* HN878–infected mice reported previously (52). Neutrophils have been shown to produce IL-10 in response to *M. tuberculosis* infection in vitro (62, 63); however, IL-10–expressing neutrophils were barely detected during *M. tuberculosis* infection in vivo as compared with other cells. Monocytes were the major innate population of IL-10^+^ cells present in the lung after *M. tuberculosis* infection, whereas macrophages, DCs, and NK cells constituted a minor proportion of IL-10–expressing cells throughout infection. Although transgenic mice overexpressing IL-10 in the macrophage/monocyte compartment exhibit enhanced susceptibility to *M. tuberculosis* infection (35), we showed in this study that specific deletion of *Il10* in these cells did not affect bacterial growth during infection. Conversely, mice unable to produce IL-10 by CD11c^+^ APCs (DCs and alveolar or interstitial macrophages) showed reduced bacterial loads in the lung at the chronic phase of *M. tuberculosis* infection compared with IL-10–competent mice, although to a much lesser extent than fully IL-10–deficient mice. This suppressive activity of CD11c^+^ APC-derived IL-10 during *M. tuberculosis* infection is in line with a recent study showing that reduced IL-10 production by APC during infection of DAP12-deficient mice correlated with increased Th1 cell activation and enhanced host protection (64).

Expression of IL-10 during *M. tuberculosis* infection was not restricted to innate cells. IL-10–expressing B cells were also detected in infected lungs, especially during the chronic phase of *M. tuberculosis* infection. However, specific deletion of *Il10* in these cells showed no effect in bacterial clearance. IL-10–expressing T cells were detected in large numbers in the lung after 3 wk of infection and, in line with a previous study reporting enhanced susceptibility to *M. tuberculosis* infection in mice overexpressing IL-10 in the T cell compartment (27), we found that mice with specific deletion of *Il10* in T cells were more resistant to *M. tuberculosis* infection than IL-10–competent mice. Despite intact IL-10 production by other cell types, the increased control of *M. tuberculosis* infection observed in the T cell–specific *Il10* mutant closely resembled the phenotype observed in complete *Il10*-deficient mice, suggesting that T cells are the critical source of IL-10 that impairs protective immune response during *M. tuberculosis* infection. The phenotype observed in the absence of T cell–derived IL-10, combined with the fact that Cre recombination can occur in ∼10% of T cells in CD11c-Cre transgenic mice (48), may suggest that partial deletion of IL-10 in the T cell compartment could at least in part contribute to the phenotype observed in CD11c-specific IL-10 knockout (*Il10^fl/fl^* CD11c-Cre^+^) mice.

Several studies have described CD4^+^ or CD8^+^ T cells as the critical source of IL-10 during protozoan and viral infection, contributing either to protection or to chronicity (50, 65–69). In this study, we show that T cells are the critical source of IL-10 during *M. tuberculosis* infection contributing to increased host susceptibility. CD4^+^ cells were the biggest population within IL-10^+^ T cells throughout infection, with Treg cells representing only a minor population of IL-10–expressing CD4^+^ T cells. Despite the potential of Treg cells to produce IL-10 during *M. tuberculosis* infection, the protective phenotype observed in T cell–specific IL-10–deficient mice is unlikely to be a result of partial loss of the regulatory function of Treg cells, because it has been previously shown that suppression of a protective immune response to *M. tuberculosis* infection by Treg cells is not dependent on IL-10 (70). CD8^+^ T cells also constituted a significant proportion of lung IL-10–expressing T cells during *M. tuberculosis* infection, in line with what has been reported postinfection of susceptible CBA/J mice with another *M. tuberculosis* strain (71). Direct ex vivo analysis of lung IL-10–expressing CD4^+^ and CD8^+^ T cells revealed that these cells were highly activated because they expressed high levels of CD44 and Tbet. In addition, lung IL-10–expressing CD4^+^ and CD8^+^ produced IFN-γ after Ag restimulation, suggesting that IL-10^+^ T cells arising in *M. tuberculosis* infection may simultaneously display effector function in addition to their regulatory activity. These findings are in agreement with previous studies showing that IL-10 produced by IFN-γ^+^ Th1 cells is critical to downregulate the immune response to other infections, such as toxoplasmosis (66), leishmaniasis (65), and malaria (50). Moreover, it has been reported that IL-10–producing *M. tuberculosis* Ag-specific T cell clones isolated from the BALF of active PTB patients (38) or from peripheral blood of tuberculin-positive individuals (72) coproduce IFN-γ, and that IL-10 production inhibits their Ag-specific proliferation and IFN-γ production (72). Taken together, our findings suggest a role for IL-10 derived from IFN-γ^+^ T cells in suppressing host-protective immune response to *M. tuberculosis* infection in mouse; these findings are supported by human studies in which IL-10 is produced by IFN-γ–producing Th1 cells.

IL-27 has been implicated in the induction of IL-10 production by Th1 cells in malaria (50) and leishmaniasis (53); however, the role of IL-27 in regulating T cell production of IL-10 during *M. tuberculosis* infection is still unclear. Our results revealed that IL-27 signaling increases *Il10* expression in lung CD4^+^ T cells early during *M. tuberculosis* infection. The expression of *Csf2* mRNA (encoding GM-CSF) in CD4^+^ T cells was significantly increased in the absence of IL-27 signaling, although similar levels of *Ifng* mRNA expression were detected in the presence or absence of IL-27R. IL-27 signaling increases susceptibility to TB (59, 60), as has been previously shown where *Il27ra^−/−^* showed a decrease in mycobacterial burden and as we also show in this study. The effect on bacterial clearance is similar to that of *Il10^−/−^* in keeping with our data that IL-27 signaling induced IL-10 and inhibited GM-CSF expression in CD4^+^ T cells during *M. tuberculosis* infection. Hence this mechanism that we propose may also contribute to the detrimental role of IL-27 during infection in addition to that suggested by others that IL-27 signaling impairs T cell fitness and protective function during *M. tuberculosis* infection (54). Similar to IL-27, type I IFN signaling was also required for maximal expression of *Il10* mRNA by lung CD4^+^ T cells during *M. tuberculosis* infection, although as we show in this article and have previously published (51, 56), there is no effect on abrogation of type I IFN signaling on bacterial load, reinforcing the complexity of type I IFN’s role in mycobacterial infection. We have previously reported that type I IFN induces IL-10 and IL-27 production by *M. tuberculosis*–infected macrophages in vitro (57). However, the effect of type I IFN on CD4^+^ T cells seems to be independent of its induction of IL-27 because similar levels of *Csf2* mRNA expression were detected in these cells in the presence or absence of type I IFN, in contrast with the increased levels detected in the absence of IL-27 signaling. Our findings suggest that both IL-27 and type I IFN signaling increase *Il10* expression in lung CD4^+^ T cells early during *M. tuberculosis* infection and that different mechanisms regulating the expression of *Il10* may be in place at early and late times postinfection.

In summary, our study has identified the cellular origins of IL-10 during *M. tuberculosis* infection in vivo and their specific contribution to host susceptibility to infection. Together our data revealed that activated effector Tbet^+^ T cells are the critical source of IL-10, accounting for increased susceptibility to *M. tuberculosis* infection. Moreover, we demonstrated that IL-27 and type I IFN signaling regulate IL-10 expression in T cells early during *M. tuberculosis* infection, providing new insights into the factors that regulate the production of IL-10 that suppresses protective immunity to TB.

## Supplementary Material

Data Supplement
