# Supplementary material for: T Cell–Derived IL-10 Impairs Host Resistance to Mycobacterium tuberculosis Infection
Source: J Immunol. 2017 Jun 5;199(2):613–23. doi: 10.4049/jimmunol.1601340 (PMC5502318; doi:10.4049/jimmunol.1601340)
Supplement: Data Supplement [file JI_1601340.zip › JI_1601340_Supplemental_Figures_1.pdf]

Supplemental Information

T cell-derived IL-10 impairs host resistance to *Mycobacterium tuberculosis* infection

Lúcia Moreira-Teixeira[1], Paul S. Redford[1], Evangelos Stavropoulos, Nico Ghilardi, Craig L. Maynard, Casey T. Weaver, Ana Paula Freitas do Rosário, Xuemei Wu, Jean Langhorne, Anne O'Garra.

[1]LMT and PSR contributed equally to this work

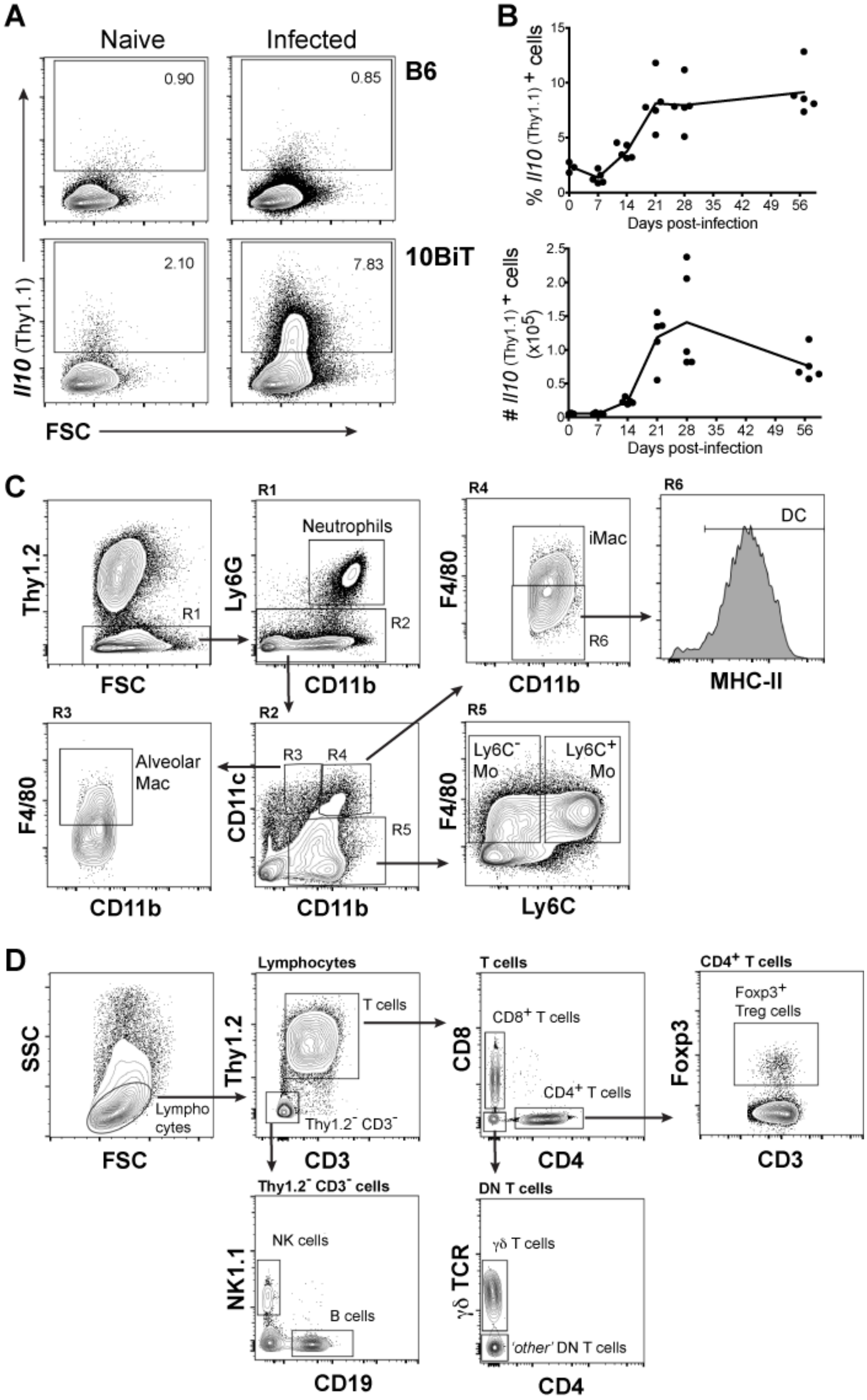

Supplemental Figure 1

Supplemental Figure 1. *Kinetic of lung IL-10-expressing cells during M. tuberculosis infection.* WT and/or 10BiT IL-10 reporter mice were infected with *M. tuberculosis* HN878. At specific days post-infection, lung cell suspensions were prepared and stained as described in Materials and Methods to detect *Il10* (Thy1.1$^+$) expressing cells. (**A**) The gating strategy after exclusion of dead cells and duplets is shown for one representative lung from naïve or infected B6 and 10BiT mice. (**B**) The percentage (top panel) and the total number (bottom panel) of total *Il10* (Thy1.1) expressing cells in the lungs at indicated time-points are shown as mean ± SEM. Results are representative of three independent experiments with individual data points depicting individual mice (3-5 mice/time-point/experiment). (**C-F**) Gating strategy for analysis of myeloid (**C**) and lymphoid cells (**D**) in *M. tuberculosis* infected lungs. Plots show concatenated data of 5 lungs at day 28 post-infection from one representative experiment. Myeloid cells: Neutrophils (Thy1.2$^-$ CD11b$^+$ Ly6G$^+$), Alveolar Macrophages (Thy1.2$^-$ Ly6G$^-$ CD11b^{int} CD11c$^+$ F4/80$^+$), Interstitial Macrophages (iMac) (Thy1.2$^-$ Ly6G$^-$ CD11b$^+$ CD11c$^+$ F4/80$^+$), DC (Thy1.2$^-$ Ly6G$^-$ CD11b$^+$ CD11c$^+$ F4/80$^-$ MHC-II$^{int/high}$) and Ly6C$^+$ or Ly6C$^-$ Monocytes (Mo) (Thy1.2$^-$ Ly6G$^-$ CD11b$^+$ CD11c$^-$ F4/80$^+$ Ly6C$^+$ or Ly6C$^-$). Lymphoid cells: T cells (Thy1.2$^+$ CD3$^+$), NK cells (Thy1.2$^-$ CD3$^-$ CD19$^-$ NK1.1$^+$) and B cells (Thy1.2$^-$ CD3$^-$ CD19$^+$ NK1.1$^-$). T cells subsets: CD4$^+$ T cells (Thy1.2$^+$ CD3$^+$ CD4$^+$ CD8$^-$), CD8$^+$ T cells (Thy1.2$^+$ CD3$^+$ CD4$^-$ CD8$^+$), gd T cells (Thy1.2$^+$ CD3$^+$ CD4$^-$ CD8$^-$ gdTCR$^+$), *'other'* DN T cells (Thy1.2$^+$ CD3$^+$ CD4$^-$ CD8$^-$ gdTCR$^-$) and Foxp3$^+$ Treg cells (Thy1.2$^+$ CD3$^+$ CD4$^+$ CD8$^-$ Foxp3$^+$).

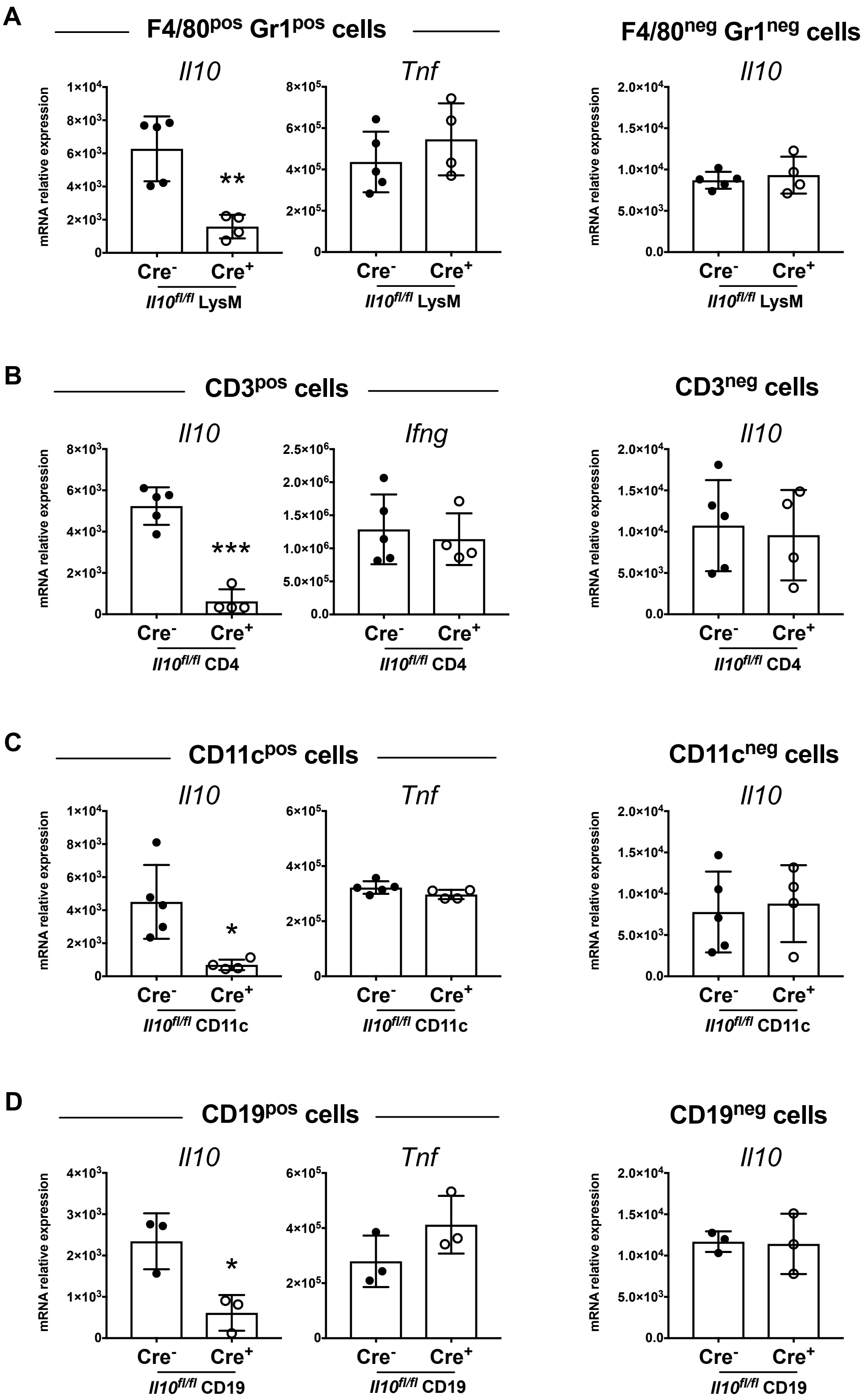

Supplemental Figure 2

Supplemental Figure 2. *Cell type-specific deletion of Il10 gene in Il10$^{fl/fl}$-Cre mice .* *Il10*$^{fl/fl}$ LysM-Cre$^+$, *Il10*$^{fl/fl}$ CD4-Cre$^+$, *Il10*$^{fl/fl}$ CD11c-Cre$^+$, *Il10*$^{fl/fl}$ CD19-Cre$^+$ mice and respective Cre- control mice were infected with *M. tuberculosis* HN878. At day 28 post-infection, lung cells from infected mice were isolated. (**A**) Lung cell suspensions from *Il10*$^{fl/fl}$ LysM-Cre$^+$ and Cre$^-$ mice were incubated with anti-F4/80 (REA12) and anti-Gr1 (RB6-8C5) biotin Abs. (**B**) Lung cell suspensions from *Il10*$^{fl/fl}$ CD4-Cre$^+$ and Cre$^-$ mice were incubated with anti-CD3 (17A2) biotin Ab. (**C**) Lung cell suspensions from *Il10*$^{fl/fl}$ CD11c-Cre$^+$ and Cre$^-$ mice were incubated with anti-CD11c (N418) biotin Ab. (**D**) Lung cell suspensions from *Il10*$^{fl/fl}$ CD19-Cre$^+$ and Cre$^-$ mice were incubated with anti-CD19 (6D5) biotin Ab. Cells were then incubated with anti-Biotin magnetic beads (Miltenyi Biotec) and positive and negative cell fractions sorted according to manufacture's instructions. *Il10* and *Tnf* or *Ifng* mRNA expression was analyzed by quantitative RT-PCR and normalized against *Hprt1* mRNA levels. Data are shown as mean ± SD of 3-5 mice per group. Differences were tested for significance by an unpaired *t* Test (**, p <0.05; **, p <0.01; ***, p <0.001*). As seen in the figure, the levels of *Il10* mRNA were significantly reduced in the respective positive cells isolated from each of the above Cre$^+$ mice compared to Cre$^-$ mice, while similar levels of *Il10* mRNA were detected in the negative cell fractions isolated from Cre$^+$ mice and respective Cre$^-$ control mice. Similar levels of other cytokines such as *Tnf* (for *Il10*$^{fl/fl}$ LysM-Cre$^+$; *Il10*$^{fl/fl}$ CD11c-Cre$^+$; *Il10*$^{fl/fl}$ CD19-Cre$^+$) or *Ifng* (for *Il10*$^{fl/fl}$ CD4-Cre$^+$) were detected in positive cells isolated from Cre$^+$ and Cre$^-$ mice. These findings demonstrate the selective deletion of *Il10* in F4/80$^+$ and Gr1$^+$ cells (macrophages, monocytes and neutrophils) in *Il10*$^{fl/fl}$ LysM-Cre$^+$ mice; CD11c$^+$ cells (mostly DC and macrophages) in *Il10*$^{fl/fl}$ CD11c-Cre$^+$ mice; CD3$^+$ cells (T cells) in *Il10*$^{fl/fl}$ CD4-Cre$^+$ mice; or CD19$^+$ cells (B cells) in *Il10*$^{fl/fl}$ CD19-Cre$^+$ mice; validating the cell-type specific deletion of *Il10* in the conditional knockout mice.

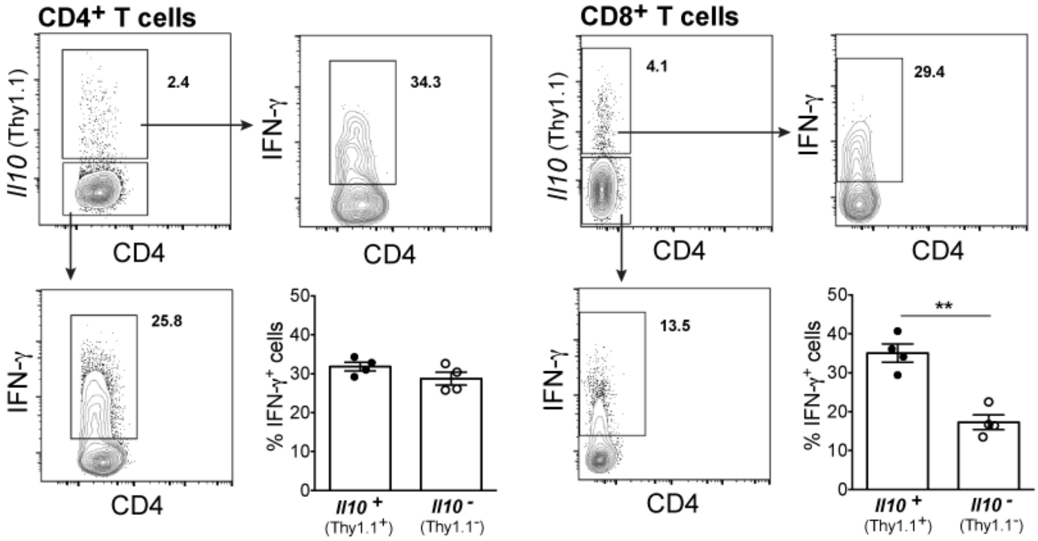

Supplemental Figure 3. *IL-10-expressing T cells from M. tuberculosis infected lungs co-produce IFN-γ upon polyclonal stimulation.* 10BiT IL-10 reporter mice were infected with *M. tuberculosis* HN878. At day 28 post-infection, lung cell suspensions were prepared and restimulated *ex vivo* with PMA (50ng/ml) plus ionomycin (500ng/ml) and Brefeldin A (10µg/ml) for 4h. Production of IFN-γ by *Il10*$^{+}$ (Thy1.1^{+}) or *Il10*$^{-}$ (Thy1.1^{-}) cells among CD4^{+} (left panel) or CD8^{+} (right panel) T cells was determined by intracellular staining. Plots show concatenated data of 4 lungs from one representative experiment out of two independent experiments (4-5 mice/ experiments). The percentage of IFN-γ expressing cells among *Il10*$^{+}$ (Thy1.1^{+}) or *Il10*$^{-}$ (Thy1.1^{-}) cells is shown as mean ± SEM, with individual data points depicting individual mice. Differences were tested for significance by an unpaired Students *t* Test (**, $p<0.01$).
